# Pharmaceutical strategies for preventing toxicity and promoting antioxidant and anti-inflammatory actions of bilirubin

**DOI:** 10.1080/14756366.2021.2020773

**Published:** 2022-01-05

**Authors:** Alessio Nocentini, Alessandro Bonardi, Simone Pratesi, Paola Gratteri, Carlo Dani, Claudiu T. Supuran

**Affiliations:** a Department of Neurosciences, Psychology, Drug Research and Child Health, Pharmaceutical and Nutraceutical Section, University of Florence, Florence, Italy; b Department of Neurosciences, Psychology, Drug Research and Child Health, Careggi University, Hospital of Florence, Florence, Italy; c Department of Neurosciences, Psychology, Drug Research and Child Health, Pharmaceutical and Nutraceutical Section, Laboratory of Molecular Modelling Cheminformatics & QSAR, University of Florence, Florence, Italy

**Keywords:** Bilirubin, hyperbilirubinaemia, jaundice, antioxidant

## Abstract

Bilirubin (BR) is the final product of haem catabolism. Disruptions along BR metabolic/transport pathways resulting from inherited disorders can increase plasma BR concentration (hyperbilirubinaemia). Unconjugated hyperbilirubinemia may induce BR accumulation in brain, potentially causing irreversible neurological damage, a condition known as BR encephalopathy or kernicterus, to which newborns are especially vulnerable. Numerous pharmaceutical strategies, mostly based on hemoperfusion, have been proposed over the last decades to identify new valid, low-risk alternatives for BR removal from plasma. On the other hand, accumulating evidence indicates that BR produces health benefits due to its potent antioxidant, anti-inflammatory and immunomodulatory action with a significant potential for the treatment of a multitude of diseases. The present manuscript reviews both such aspects of BR pharmacology, gathering literature data on applied pharmaceutical strategies adopted to: (i) reduce the plasma BR concentration for preventing neurotoxicity; (ii) produce a therapeutic effect based on BR efficacy in the treatment of many disorders.

## 1. Introduction

**Figure 1.**
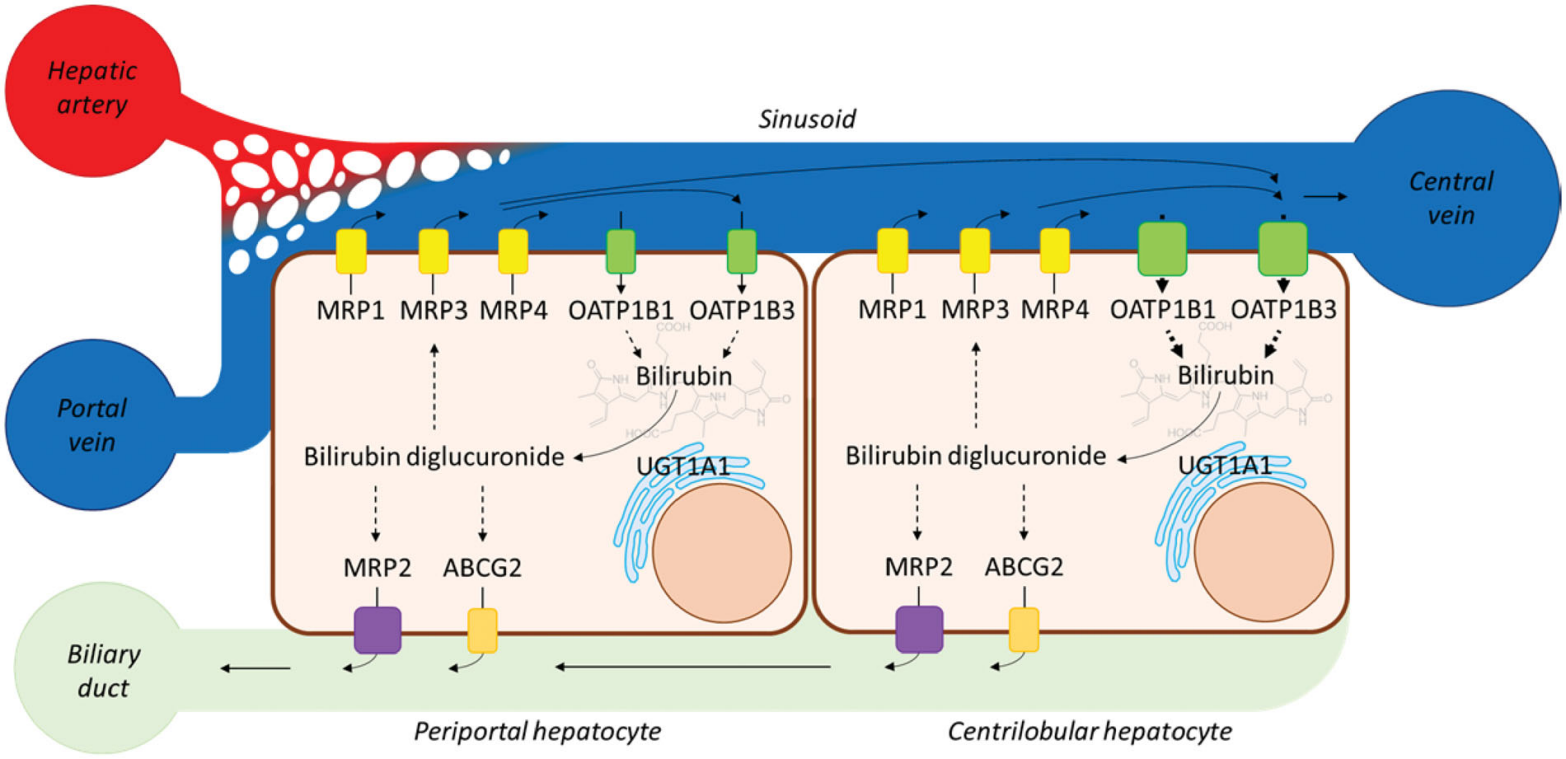
Bilirubin excretion pathway. MRP1, MRP3, MRP4, OATP1B1, and OATP1B3 are sinusoidal unconjugated and conjugated bilirubin transporters, whereas MRP2 and ABCG2 are implicated in its elimination in the biliary duct.

Bilirubin (BR) is a highly hydrophobic, water-insoluble tetrapyrrole derivative representing the end product of haem breakdown. About 80% derivatives from erythrocyte haemoglobin degradation in the reticuloendothelial system, while the remaining 20% comes from inefficient erythropoiesis in bone marrow and degradation of other haem proteins (e.g. myoglobin, cytochromes, catalases)^1–4^. In plasma, unconjugated bilirubin (UCB) binds to albumin and is transported to the liver, up taken by passive transmembrane diffusion combined with active transport mediated by several sinusoidal transporters (i.e. sinusoidal membrane-bound organic anion transporting polypeptides OATP1B1 and OATP1B3)^1^^,^^5^. In hepatocytes, BR binds to ligandin and is transported to endoplasmic reticulum where the enzyme uridine diphosphate glycosyltransferase 1A1 (UGT1A1) catalyses its conjugation with glucuronic acid^1^^,^^6^. Therefore, the excretion of conjugated bilirubin into bile (biliary duct) is mediated by the ATP-dependent transporter MRP2 (identified as a multidrug resistance-associated protein) and in part by ATP-binding cassette efflux transporter ABCG2^5–7^. A substantial fraction of bilirubin conjugates is primarily secreted by the ATP-dependent transporter MRP3 at the sinusoidal membrane into the blood and is subsequently reuptaken by OATP1B1 and OATP1B3, whose expression is higher in centrilobular hepatocytes (Figure 1)^1^^,^^2^. This transfer process (hopping) of conjugated bilirubin and other substrates from the periportal to the centrilobular zone of the liver lobule is thought to protect periportal hepatocytes against elevated concentrations of various xenobiotics^2^.

Disruptions along these metabolic/transport pathways can cause an increase in unconjugated bilirubin (e.g. from increased red blood cell destruction or impaired bilirubin conjugation) or conjugated bilirubin (e.g. from hepatocellular damage or biliary tract obstructions)^1^^,^^8^. Hyperbilirubinemia is traditionally defined as serum BR concentrations above 1 mg/dL, while jaundice (i.e. yellow discoloration of skin and sclera of eyes) usually occurs at serum concentration above 2–3 mg/dL^9–12^. Depending on the cause, hyperbilirubinaemia may appear at birth or at any time afterwards. Unconjugated hyperbilirubinaemia may cause the lipid-soluble BR to accumulate in brain, potentially causing seizures and irreversible neurological damage, leading to a condition known as acute bilirubin encephalopathy (ABE) and kernicterus spectrum disorders (KSDs), with devastating, permanent neurodevelopmental handicaps or exitus^13–19^.

BR deposited in the brain produces toxic effect by inhibition of DNA synthesis, uncoupling of oxidative phosphorylation and inhibition of adenosine triphosphatase (ATPase) activity in brain mitochondria^13–16^. BR impairs several enzymatic systems, RNA and protein synthesis in brain and liver. Further toxic effects of BR result from alteration of carbohydrate metabolism in the brain^17–19^.

Depending on the exposure level, UCB hyperbilirubinaemia can produce from unnoticeable effects up to severe brain damage and even death. Newborns are especially vulnerable to hyperbilirubinemia-induced neurological damage^9^. Hence, it is crucial to carefully monitor newborns for alterations in their serum BR levels. Relevantly, yellow discoloration observed in 60% of term and 80% of preterm infants usually disappears spontaneously^10^.

Three types of inherited, predominantly unconjugated non-haemolytic hyperbilirubinaemia have been identified according to different levels of UGT1A1 activity, related to protein mutations^1^^,^^20^: Crigler–Najjar syndrome type I (CN1), Arias syndrome type II (CN2) and Gilbert's syndrome (GS). On the other hand, two types of hereditary conjugated jaundice exist, that are the benign Dubin–Johnson and Rotor syndromes^21^^,^^22^.

**Figure 2.**
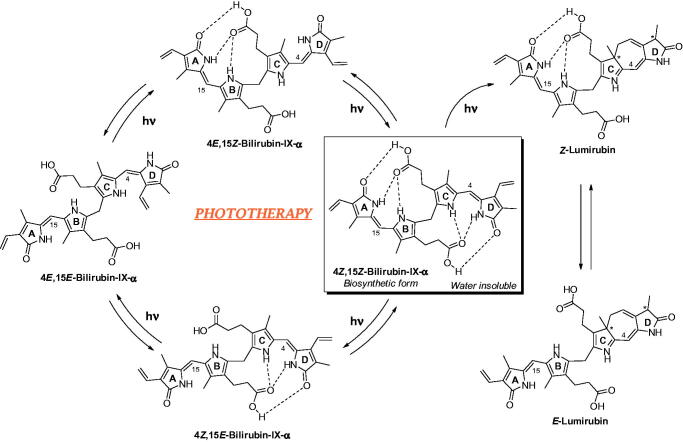
Configurational photoisomerization of (4Z,15Z)-bilirubin (biosynthetic and water-insoluble form) to (4Z,15E)-, (4E,15Z)- and (4E,15E)- isomers. Structural photoisomerization of (4Z,15Z)-bilirubin to (Z)-lumirubin and configurational isomerisation of the latter to (E)-lumirubin are also shown. Black dashed lines represent intramolecular hydrogen bonds.

CN1, the most deleterious form, shows the complete or almost complete absence of UGT1A1 enzyme activity with severe jaundice^23^^,^^24^. Jaundice occurring immediately after birth can be complicated by ABE and KSDs, that, before the introduction of phototherapy and blood exchange transfusion, was fatal in almost all cases or caused serious brain damage with permanent neurologic sequelae. Phototherapy produces the photoisomerization of unconjugated BR biosynthetic (and water-insoluble) form, that is 4Z,15Z-Bilirubin-IX-α, to its water-soluble photoisomers and their successive elimination via bile (Figure 2)^11^^,^^25^^,^^26^. The effectiveness of phototherapy decreases gradually with age and patients are at higher risk of sudden brain damage^26^. New treatments, such as hepatocyte or hepatic progenitor cell transplantation, are been used^27^. However, liver transplantation is still considered to be the only definitive treatment for CN1^1^^,^^26^.

CN2 is characterised by reduced UGT1A1 enzyme activity with a moderate degree of nonhemolytic jaundice. Bilirubin levels are not critical and CN2 is only rarely complicated by KSDs^28^^,^^29^.

GS is characterised by fluctuating mild, unconjugated non-haemolytic hyperbilirubinaemia usually diagnosed around puberty, and aggravated by concomitant illness, stress, fasting or after administration of certain drugs^30^. GS is the most frequent hereditary jaundice affecting nearly 5–10% of the Caucasian population^31^. GS is benign and carriers present with no liver disease^32^.

Additionally, neonatal non-haemolytic jaundice can be the result of a higher rate of foetal red blood cells (RBCs) turnover than that is seen in adults. In contrast, haemolytic jaundice results from immunological breakdown of RBCs by maternal antibodies targeting blood group antigens^33^.

**Figure 3.**
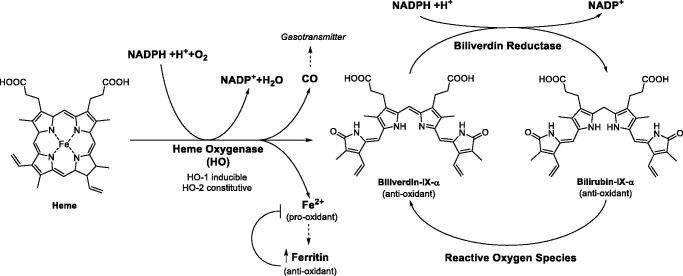
Bilirubin biosynthetic pathway and antioxidant mechanism.

On the other hand, it is extremely relevant that BR is an endogenous substance which acts as an antioxidant and anti-inflammatory agent in the serum^34–38^. Haem oxygenases (HOs, chiefly isoform 1) degrades haem-producing biliverdin, carbon monoxide (a gasotransmitter and efficient anti-inflammatory), a Fe(II) ion and consuming oxygen and a reducing agent, NADPH (Figure 3). BR derives from the reduction of biliverdin by biliverdin reductase (BVR). BR is able to scavenge singlet oxygen with high efficiency, to react with superoxide anions and peroxyl radicals, and to serve as a reducing substrate for peroxidases in the presence of hydrogen peroxide or organic hydroperoxides. Acting as a reducing agent, BR oxidises back to biliverdin, which is recovered again to BR by BVR by a cycle that detoxifies up to 10000 times the oxidant excesses (Figure 3)^35^. It was shown that BR exerts antioxidant and cytoprotective functions in a physiologically complementary manner with glutathione^37^. There is evidence of the protective effect of mild to moderately elevated levels of BR against diseases related to oxidative stress^38^. In fact, moderately higher BR levels are positively associated with reduced risk of cardiovascular disease, Alzheimer’s disease, ischemia-reperfusion, diabetes, respiratory disease, cancer, metabolic syndrome and obesity, suggesting that increased BR production is an adaptive response against oxidation^36–46^. Further, BR demonstrated experimentally immunosuppressor effects^47^. Induction of the HO 1/biliverdin/bilirubin system decreases episodes of acute and chronic rejection in solid organ transplants^48^.

While the role of BR as a scavenger of reactive oxygen species is well documented *in vitro* and animal studies, in the context of newborn hyperbilirubinaemia the role of BR in balancing antioxidant and pro-oxidant agents is still debated, because of contradictory results^49^. In fact, in a recent literature survey, high BR levels have been associated with an increase of oxidative stress both in term and preterm infants^50–52^. However, it is evident that factors other than BR are the most likely cause of this increase: (i) the release of redox-active iron from haem by HO 1 might induce an increase of oxidative stress because infants lack of an adequate induction of ferritin to effectively remove iron; (ii) the prooxidant effect of phototherapy adopted to reduce hyperbilirubinemia^53^^,^^54^.

Both faces of BR pharmacology, harmful at very high level and efficient antioxidant at moderately elevated levels, are subject of research in the biomedical field. The present review gathers and discusses the literature data over transport, removal, complexation and controlled release of BR.

## 2. The lipophilic bilirubin binds specific proteins in plasma and cellular media

The biosynthetic form of BR (4*Z*,15*Z*-bilirubin-IX-α; Figure 2) is insoluble in water at neutral pH^55^. Thus, BR binds serum albumin in blood plasma to reach the liver. The lipophilicity and poor aqueous solubility of bilirubin were long considered a paradox, since the molecule is a dicarboxylic acid containing several polar functional groups. The crystal structure of the compound revealed that the molecule adopts a “ridge-tile” conformation in which the interplanar angle between the dipyrrin one groups is about 100°. Each of the two propionic acid groups establishes three H-bonds with the opposite dipyrrinone ring disabling interactions with water molecule (Figure 2)^55^. The ridge-tile conformation also occurs in solution where, however, BR can flip between two enantiomeric conformers (P and M), both of which maintain the internal H-bond network. Phototherapy, that is used to treat hyperbilirubinaemia in newborns, transforms BR in its water-soluble photoisomers, whose polar functional groups are able to form polar contacts with water molecules (Figure 2).

**Figure 4.**
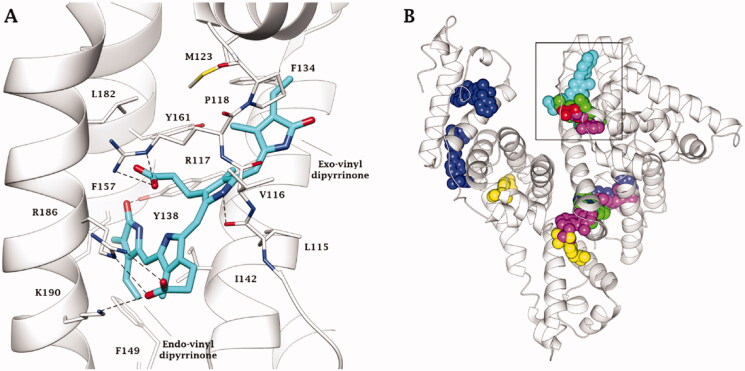
A) X-ray solved structure of 4Z,15E-bilirubin-IX-α in complex with HSA (PDB 2VUE). B) Superposition of BR (cyan), naproxen (red, PDB 2VDB), indomethacin (green, PDB 2BXM), diclofenac (magenta, PDB 4Z69), ibuprofen (yellow, PDB 2BXG) and sulfasalazine (blue, PDB 6R7S) bound to HSA. H-bonds are depicted as black dashed lines. Amino acids are labelled with one letter symbols: F, Phe; I, Ile; K, Lys; L, Leu; P, Pro; R, Arg; V, Val; Y, Tyr.

The binding mode of UCB to human serum albumin (HAS) was revealed by X-crystallography, performed in dark conditions to avoid photoisomerization. Nonetheless, a significant predominance of the photometabolite 4*Z*,15*E*-bilirubin-IX-α was identified in the electron density of the ligand bound to the protein. The structure was deposited in the protein data bank (PDB) with the code 2VUE and a resolution of 2.42 Å (Figure 4(A)). One carboxylic function of the ligand engages a salt bridge and H-bonds with the side chain guanidine of R117, while the other carboxylate forms analogue interaction with the positively charged groups of R186 and K190 side chains. The NH group of ring B is involved in a H-bond with the backbone C = O of L115. The endo-vinyl dipyrrinone ring accommodates in a lipophilic cleft lined by I142, F149 and F157, while the C = O moiety is in H-bond distance with the sidechain OH of Y138. The exo-vinyl dipyrrinone ring is in contacts with residues P118, M123, F134 and Y161, whereas the amide group are exposed to the solvent (Figure 4(A)).

Modelling studies were reported for the biosynthetic 4*Z*,15*Z*-bilirubin-IX-α and HSA and indicate an overall binding conformation that is broadly similar to the crystallographic pose adopted by the 4*Z*,15*E* isomer, though some binding inconsistencies remain when related to CD measurements^55^.

It is relevant that in the bloodstream nonsteroidal anti-inflammatory drugs (NSAIDs) were shown to be mostly bound to HAS^56^. Crystallographic analysis showed that naproxen, ketoprofen, diclofenac and indomethacin even bind HSA in the same binding site pointed out for BR, whilst ibuprofen binds in different regions of the plasma protein (Figure 4(B))^57^. However, it was shown in solution that ibuprofen binds plasma albumin interfering with the binding of BR in jaundiced newborn infants, where ibuprofen is used for closing the ductus arteriosus^58^. In fact, data from a 2010 research paper by Soligard et al. indicate that ibuprofen is a competitive displacer of BR from albumin *in vitro* increasing the level of unbound plasma BR, as such as sulphisoxazole, a drug that causes KSDs in premature infants^59^. In this context, a binding site competition between ibuprofen and BR was detected *in silico* by Evoli et al. upon a thorough absolute binding free energy calculations^60^. Additionally, Paar et al showed by a structural in solution visualisation of albumin dynamicity that the binding, transport, and release of its cargos strongly depend on albumin highly flexible conformation, which is affected by bound ligands induced by physiological and pathological conditions^61^. Berns et al. also demonstrated that BR reduces neuron viability in rat cortical neuronal culture and ibuprofen increases this effect^62^. In contrast, in a successive study, Desfrere et al. determined no correlation between unbound BR and ibuprofen concentrations in preterm neonates during ibuprofen treatment of patent ductus arteriosus. The authors thus concluded that the drug does not displace BR from HSA in preterm infants, but caution should be applied in cases of elevated BR concentration or when higher doses of ibuprofen are ad-ministered^63^.

It is of interest that α-fetoprotein (AFP) is the foetal analogue of serum albumin and functions as a carrier protein for bilirubin^64^. AFP is one of the major plasma glycoproteins in the early foetal stage. Moreover, AFP is used as a biomarker for maternal testing for foetal screening indicating problems in the foetus, placenta or liver diseases in the woman. AFP is also a marker for certain tumours, since it reappears in serum in cases of hepatoblastoma, and hepatocellular carcinoma^65^.

Upon entrance within the hepatocytes, BR is transported to the smooth endoplasmic reticulum for glucuronidation by binding to two cytosolic proteins: Z protein (fatty acid-binding protein) and ligandin (Y protein)^66^^,^^67^. In normal conditions, ligandin is probably the principal hepatic bilirubin-binding protein and may serve the same protective and transport functions intracellularly as does albumin in plasma. It also limits reflux of bilirubin into plasma, since its affinity for bilirubin is at least five times greater than that of albumin. Z protein becomes important at high plasma bilirubin concentrations. The concentration of ligandin in the liver does not reach adult levels until several weeks after birth, whereas neonatal and adult levels of Z protein are the same. This lack of ligandin, together with low hepatic glucuronyltransferase activity, is the probable cause of transient, “physiological,” nonhemolytic, neonatal jaundice^66^^,^^67^.

## 3. Bilirubin transporters across biological membranes

In adults, 200–300 mg of bilirubin are produced daily, as a result of the physiologic turnover of haemoglobin and cellular cytochromes^1^^,^^2^^,^^68^. Surprisingly, liver takes up 0.13–0.2 mg of BR *per* minute. A thoroughgoing review on membrane transporters of BR and its conjugates was recently published^2^. The latter systematically gathered data over the mechanisms of BR uptake into the liver, excretion into the bile, reflux from the liver to the blood, and barrier for BR transport into the brain. As mild increases are related to reduction of cardiovascular disease risk, type-2 diabetes mellitus and other conditions, the authors deemed important to understand if membrane transporters may participate in these beneficial fluctuations. The outcome of the study pointed out 11 electrochemical potential-driven and 8 primary active membrane transporters (listed in Table 1)^68–72^. The authors concluded that “paradoxically, the remarkable advancements in this field have only confirmed the elusive mechanism enabling UCB to diffuse into the liver as if no cellular boundary existed.”

**Table 1.** Membrane transporters of bilirubin and its conjugates^2^.

Acronym	Organism	Names and synonyms	HGNC	UCB/conjugated BR
**OABP**	Rat	Organic Anion Binding Protein	–	UCB
**BBBP**	Rat	Bilirubin Binding protein	–	UCB
**BTL**	Human, rat	Bilirubin transporter	–	UCB
**Oatp1a1**	Mouse, rat	Organic anion-transporting polypeptide 1a1	SLCO1A2	UCB, conjugated BR
**Oatp1a4**	Mouse, rat	Organic anion-transporting polypeptide 1a4	SLCO1A2	UCB, conjugated BR
**Oatp1a5**	Mouse, rat	Organic anion-transporting polypeptide 1a5	SLCO1A2	UCB, conjugated BR
**Oatp1a6**	Mouse	Organic anion-transporting polypeptide 1a6	SLCO1A2	UCB, conjugated BR
**Oatp1b2**	Mouse, rat	Organic anion-transporting polypeptide 1b2	SLCO1B2	Conjugated BR
**OATP1B1**	Human	Organic anion-transporting polypeptide 1B1	SLCO1B1	UCB, conjugated BR
**OATP1B3**	Human	Organic anion-transporting polypeptide 1B3	SLCO1B3	UCB, conjugated BR
**OATP1A2**	Human	Organic anion-transporting polypeptide 1A2	SLCO1A2	UCB, conjugated BR
**Mdr1a**	Mouse, rat	Multiple drug resistant protein 1a	ABCB1	UCB
**MDR1**	Human	Multiple drug resistant protein 1	ABCB1	UCB
**MRP1** **Mrp1**	Human mouse, rat	ATP-binding cassette sub-family C 1a	ABCC1	UCB
**MRP2** **Mrp2**	Human mouse, rat	ATP-binding cassette sub-family C 2	ABCC2	Conjugated BR
**MRP3** **Mrp3**	Human mouse, rat	ATP-binding cassette sub-family C 3	ABCC3	Conjugated BR
**MRP4** **Mrp4**	Human mouse, rat	ATP-binding cassette sub-family C 4	ABCC4	None
**MRP5** **Mrp5**	Human mouse, rat	ATP-binding cassette sub-family C 5	ABCC5	UCB

## 4. Pharmaceutical strategies applied to reduce bilirubin serum concentration

The two most common treatments for infant jaundice are phototherapy and exchange transfusion^73^. Other serious liver diseases (e.g. acute liver failer) dramatically reduce the liver ability to remove waste products such as albumin, leading to further damages in patients^74^. The application of hemoperfusion (i.e. a method of filtering the blood extracorporeally to remove a toxin) for the removal of BR has been also pursued for over 30 years to reduce the risk associated with exchange transfusion (e.g. hypoglycaemia, hypocalcaemia, acidosis, coagulopathies, graft-versus host disease, transmission of infectious diseases)^75^. In this context, several adsorption technologies have been suggested and applied in clinical treatment as extracorporeal methods to remove BR from blood, such as activated charcoal, ionic exchange resins, adsorptive membranes and some polymeric adsorbents^76^. However, the application of such first materials has been limited by their poor biocompatibility, lack of specificity (undesired removal of essential compounds as thyroxine, cortisol, and aldosterone) and low capacity^77^. The development of bilirubin adsorbents with excellent mechanical properties, adsorption performance and hemocompatibility is still a considerable challenge.

**Figure 5.**
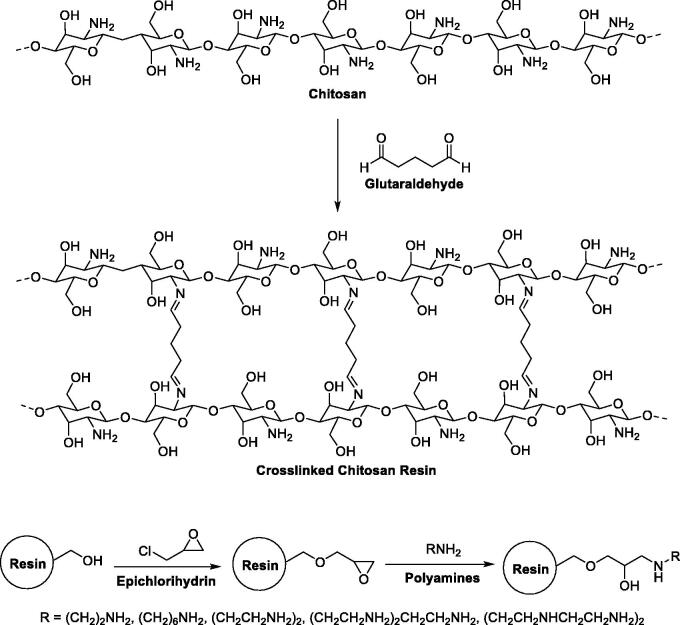
Production pathway for crosslinked chitosan spherical beads^77^.

For instance, crosslinked chitosan (CS) resins have been proposed to achieve bilirubin caption because of their blood compatibility and antifungal activity^78^. In 2000, Yu et al. obtained and optimised crosslinked chitosan spherical beads by a crosslinking reaction of CS with glutaraldehyde (Figure 5)^77^. The resins were thus activated via epichlorohydrin and functionalised with varied polyamines (i.e. di-, tri-, tetra- and pentamines). Adsorption studies were carried on the protonated resin forms and evaluated the adsorption capability of BR in a phosphate buffer solution (pH 7.4). Most spherical beads produced an effective BR adsorption with both electrostatic interactions (between BR and the positively charged amine groups of the resin) and hydrophobic interactions (between BR and the polyamine hydrophobic spacers) being the driving force for the adsorption. The functionalised crosslinked CS resins showed higher adsorption capacity compared with simple crosslinked CS resin for both unconjugated and conjugated bilirubin. When evaluated with dog blood, the resins showed a good biocompatibility that gives crosslinked CS beads a certain advantage over activated charcoal and other polymeric adsorbents.

In 2015, Wei et al. prepared heparin-modified chitosan/graphene oxide hybrid hydrogel (hepCS/GH) for bilirubin adsorption using a lyophilization–neutralization–modification strategy^79^. These hybrid hydrogels displayed a unique foam-like porous structure and excellent mechanical flexibility. The incorporation of graphene oxide into the chitosan matrix enhanced both mechanical features BR adsorption capacity. It was demonstrated that hep-CS/GH successfully competed with albumin, and could effectively adsorb bilirubin from a bilirubin-enriched serum. After the hydrogel was modified with heparin, protein adsorption, platelet adhesion and haemolysis were reduced, and the plasma clotting time was prolonged indicating a superior hemocompatibility of hep-CS/GH.

Another chitosan matrix improved for BR adsorption was proposed by Chen et al. in 2019^80^. Cross-linked chitosan/SiO_2_-loaded graphene (CS/graphene-SiO2) composite beads were achieved by a phase-inversion method. Adsorption results demonstrated that CS/graphene-SiO_2_ beads possessed much better adsorption capacity for BR compared to pure CS beads, mainly due to the synergistic effect between the hydrophobic forces of graphene and the electrostatic interactions provided by the amino groups of CS. Again, the mechanical strength of composite beads was significantly improved as compared to simple CS beads, due to the incorporation of graphene. The effects on haemolytic activity and the components of blood were negligible, indicating an excellent compatibility with blood.

In 2018, Li et al instead used CS as a reinforce in macroporous reduced graphene oxide aerogels (GO/CS)^81^. The latter showed a high adsorption capacity and a fast adsorption rate. Moreover, the low haemolysis ratio and negligible anticoagulant activity of rGO/CS aerogels suggested good blood compatibility.

In 2009, Ando et al. fabricated carbon nanotube sheets for BR adsorption^82^. They showed that multi-walled carbon nanotubes (MWCNTs) exhibit greater BR adsorption capacity than of single-walled carbon nanotubes (SWCNTs). To guarantee the safety of the adsorbents, the authors fabricated carbon nanotube sheets in which leakage of CNTs to the plasma is suppressed. Since SWCNTs are more robust sheets, a complex system consisting of SWCNTs as the scaffold and MWCNTs as the adsorbent was proposed and claimed as promising for an adsorbent material for plasma apheresis.

It should be noted that CS salts were also proposed in a 1981 patent as active ingredient for an oral preparation for treating hyperbilirubinemia^83^. Finely ground CS was suspended in a liquid carrier, preferentially fruit juice. The rational of the invention is that CS is nondigestible and passes through the whole digestive tract, adsorbing conjugated BR secreted with bile or the UCB freely accumulating within the intestinal lumen. To the best of our knowledge, no successive such strategies were reported in literature.

Cyclodextrins (CDs) are cup-shaped oligosaccharides formed by α-(1,4)-linked glucose units. α-, β- and γ-CD with different cavities can include “guest” molecules of suitable sizes form inclusion forming complexes^84^. A first attempt to complex BR with CDs and non-cyclic oligosaccharides was reported by Kano in 1988^85^. As BR can adopt enantiomeric conformations with R- and S-helices via intramolecular hydrogen bonding the authors studies the mechanism for enantioselective binding of BR to cyclic and non-cyclic oligosaccharides via circular dichroism.

**Figure 6.**
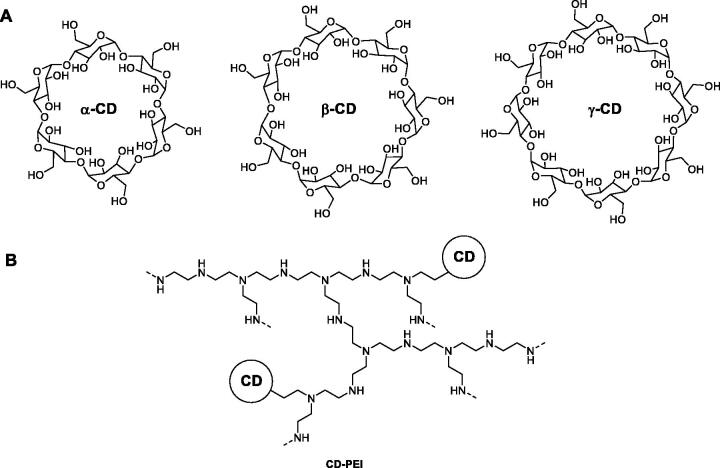
Synthetic pathway for the production of CD-grafted polyethyleneimine as water-soluble adsorbent for BR removal^86^.

In 2012, Wang and co-workers prepared three water-soluble adsorbents for removing plasma BR by separately coupling α-, β- and γ-CDs to branched polyethyleneimine (PEI), that is a highly reactive water-soluble polymer (Figure 6)^86^. PEI was shown to be good matrix for water-soluble adsorbent because of the branched structure and large number of primary amine groups. The BR bilirubin-binding capacity of each adsorbent was evaluated both in bovine serum albumin (BSA) solution and in plasma obtained from a patient with liver disease. Dialysis experiments suggested β-CD-PEI had the strongest bilirubin-binding capacity, even higher than BSA under the same condition. Molecular docking study showed that BR showed the strongest interaction with β-CD both in the 1:1 and 1:2 ratio. The 1:2 ratio complex was the most favourable in terms of binding energy. The latter was deemed to be feasible in CD-PEI adsorbents because, unlike solid adsorbents, are not cross-linked allowing couples of CDs to adopt the best binding with BR.

A new haemoadsorption device was reported in 2019 by Gemelli et al.^87^. Cytosorb® consists of an innovative porous polymer beads adsorption system, shown to be biocompatible and haemocompatible. Cytosorb® was shown to irreversibly adsorb and remove albumin-bound BR breaking the strong bond of the complex with negligible albumin loss. It is also specifically approved for the removal of cytokines, and myoglobin. In regards to cytokine removal, Cytosorb® was reported to reduce cytokines to levels that are no longer toxic, while still keeping the immune system intact.

A different strategy was adopted by Lavin and co-workers in 1985 to reduce serum BR concentrations^88^. They built a small blood filter containing immobilised bilirubin oxidase (BOX) from *Myrothecium verrucaria*, able to degrade in a single pass more than 90% of the BR from human or rat blood.

**Figure 7.**
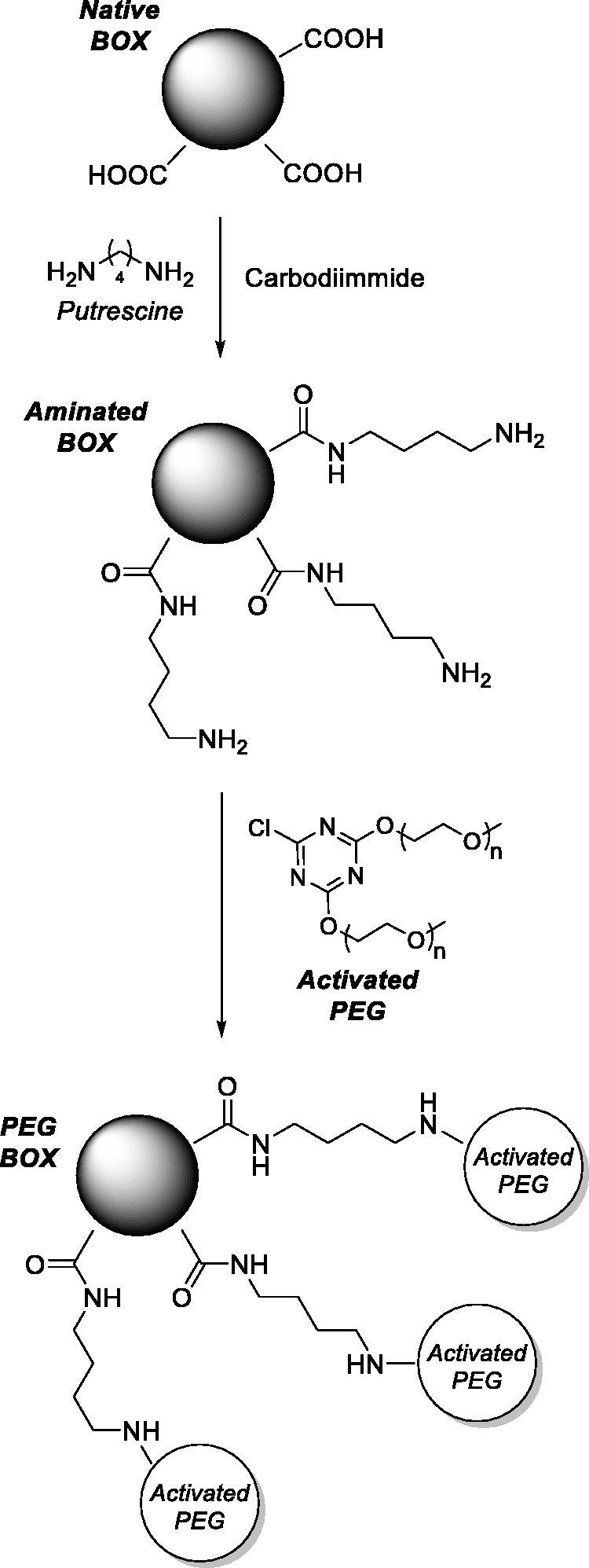
Synthetic approach adopted to attach PEG to BOX^89^^,^^90^.

Few years later Kimura et al. deemed such BOX column system to have many inconveniences, such as physical limitations, clotting problems, and infections^89^^,^^90^. The authors modified BOX with polyethylene glycol (PEG) to increase its plasma half-life (Figure 7), to diminish its immunogenicity, and to make it injectable for at least a limited time when patients are at a critical stage. This strategy was demonstrated to be effective therapeutically in experimentally jaundiced rabbits and rats. In fact, the activity of native BOX rapidly decreased after intravenous injection, whereas that of PEG-BOX decreased more slowly and was detectable even 48 h after injection.

A more complex mechanism for BR removal was proposed by Sarkar et al in 2012, that is ZnO nanoparticle (NP)-sensitized BR degradation via defect-mediated nonradiative energy transfer pathway^91^. The authors reported efficient photodegradation of surface adsorbed BR on the ZnO NPs. However, no evaluation with blood was performed.

**Figure 8.**
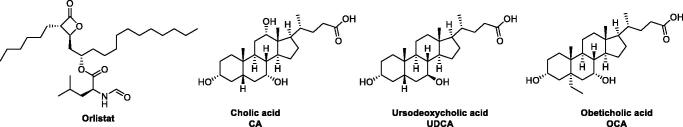
Drugs administered *per os* to attain the reduction of BR serum concentration.

Other strategies based on drug therapy were proposed to attain the reduction of BR serum concentration. In 2003, Cuperus, Verkade and co-workers et al. initially demonstrated in Gunn rats that dietary supplementation with the lipase inhibitor orlistat (Figure 8) decreased plasma UCB levels, parallel to an increase in faecal fat excretion^92^. Orlistat treatment was equally effective as continuous phototherapy in Gunn rats, and combined treatment was more effective than either treatment alone. In 2006, the same authors investigated the mechanisms underlying such hypobilirubinaemic effects and showed that orlistat and phototherapy lower plasma bilirubin concentrations in Gunn rats by increasing (net) intestinal influx of UCB, either by transmucosal excretion (orlistat), or increased biliary secretion (phototherapy)^93^.

In 2009, again Cuperus et al. implemented their studies demonstrating that gastrointestinal transit time and plasma UCB concentrations are linearly related in Gunn rats^94^. Hence, the authors showed that acceleration of the gastrointestinal transit by the laxative polyethylene glycol (PEG) decreases plasma UCB concentrations in hyperbilirubinaemic Gunn rats. The results indicate that the hypobilirubinaemic effect of PEG treatment was due to a selective increase in transmucosal UCB diffusion. PEG treatment decreased plasma UCB more rapidly than did orlistat, a well-known experimental oral treatment strategy for unconjugated hyperbilirubinaemia. Importantly, the combination of phototherapy with PEG resulted in a therapeutic efficacy not only superior to single PEG treatment, but also to treatment combinations that were explored in comparable Gunn rat experiments. It is noteworthy that numerous clinical trials with PEG have shown an absence of serious side effects, and a milder side effect profile compared with other laxatives^95^.

In the same year, the same research team tested the hypothesis that oral administration of bile salts (Figure 8), known to increase the UCB biliary excretion of UCB, decreases unconjugated hyperbilirubinemia in a Gunn rat model^96^. Animals were fed a standard diet or the same diet supplemented with ursodeoxycholic acid (UDCA) or cholic acid (CA). The authors showed that dietary bile salt administration induces a large and persistent decrease in plasma UCB concentrations in the animal model. In particular, both UDCA and CA enhance UCB turnover by increasing its faecal disposal.

In a 2021, research paper van der Schoor and co-workers investigated instead the potential of therapeutic bile acids UDCA and obeticholic acid (OCA, 6-α-ethyl-CDCA), a farnesoid-X-receptor (FXR) agonist (Figure 8), as preventive treatment options for neonatal hyperbilirubinaemia^97^. The adopted animal models were hUGT1*1 humanised mice and Ugt1a-deficient Gunn rats. hUGT1*1 mice are transgenic for the entire human UGT1A locus. Both UDCA and OCA effectively decrease plasma BR in a humanised mouse model of neonatal hyperbilirubinemia. Both compounds also significantly reduced brain levels of bilirubin in these mice. These effects were shown to depend at least partially on induction of UGT1A1 expression in intestine (and not in liver) and on FXR activation. In fact, by the Ugt1a-deficient Gunn rat model, UDCA but not OCA significantly decreases plasma bilirubin, indicating that at least some of the hypobilirubinaemic effects of UDCA are independent of UGT1A1. Being both UDCA and OCA are FDA-approved drugs, and UDCA even already approved for neonatal use, they should be readily available drugs for clinical studies.

## 5. Applied pharmaceutical strategies to promote bilirubin antioxidant and anti-inflammatory action

The other main research area over BR focuses on the accumulating evidence of the health benefits induced by BR an antioxidant, anti-inflammatory, immunomodulatory, and anti-apoptotic action^34^. Several epidemiological studies indicate that individuals with higher levels of total serum bilirubin experience a lower risk of certain cardiovascular diseases and cancers, among other disorders. In particular, individuals with GS experiencing hyperbilirubinemia in response to certain stressors appear to be protected from all-cause mortality, with progressively elevated total BR associated with protection from ischaemic heart and chronic obstructive pulmonary diseases^98^. In fact, BR reduces circulating cholesterol, oxidative lipid/protein modifications, and blood pressure. In addition, BR inhibits platelet activation and protects the heart from ischaemia-reperfusion injury (IRI)^99^^,^^100^. These effects attenuate multiple stages of the atherosclerotic process in addition to protecting the heart during resultant ischaemic stress.

**Figure 9.**
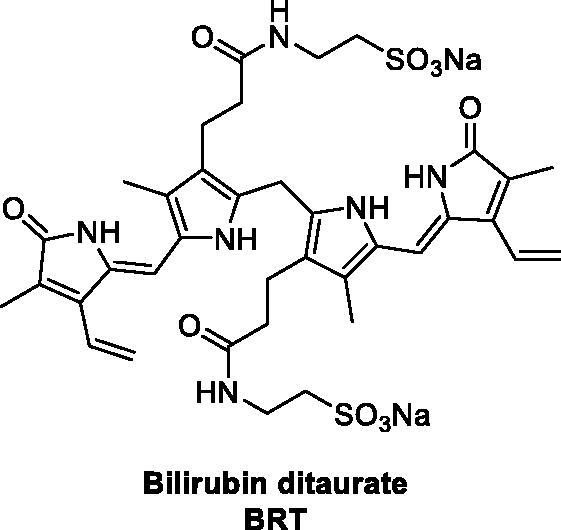
Structure of bilirubin ditaurate (BRT)^101^.

In this context, based on their previous finding that BR may impair both inappropriate platelet activation and ROS generation during storage, Bulmer and co-workers investigated the anti-platelet effects of a water-soluble BR analogue, that is, bilirubin ditaurate (BRT, Figure 9)^101^. By the use of agonist induced platelet aggregation, dense granule exocytosis and flow cytometric analysis, the authors showed acute BRT platelet exposure inhibited platelet activation and reduced platelet ROS production *ex vivo*.

**Figure 10.**
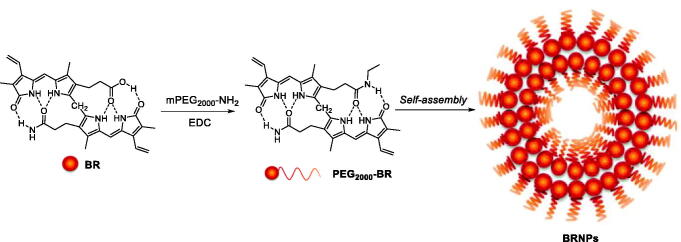
Synthesis of PEG-BR and formation of BRNPs upon self-assembly^102^.

In 2016, Lee et al. proposed BR-based nanoparticles to overcome the critical solubility issue of BR and to be a therapeutic nanomedicine in various inflammatory diseases owing to the ability of BR to scavenge a variety of ROS and modulate the immune responses^102^. PEG was covalently attached to bilirubin, yielding PEGylated bilirubin (PEG-BR) (Figure 10). The PEG-BR self-assembled into nanoscale particles (BRNPs), that were highly efficient hydrogen peroxide scavengers, thereby protecting cells from H_2_O_2_-induced cytotoxicity. In a murine model of ulcerative colitis, intravenous injection of BRNPs showed preferential nanoparticles accumulation at the sites of inflammation and significantly inhibited the progression of acute inflammation in the colon. The authors also showed that BRNPs can be important immunomodulatory agent useful as a therapeutic for allergic lung inflammatory disease^103^.

In a parallel study, the same authors showed that exposure of BRNPs to either ROS or external laser light causes rapid disruption of the BRNP nanostructure as a result of a switch in BR solubility. In fact, both BR photoisomers, resulting from external light (e.g. phototherapy) and biliverdin, resulting from ROS exposure, exhibit significantly increased water solubility. The authors suggested that this BRNP disruption might be exploited to release drugs encapsulated in the nanostructures^104^. BRNPs loaded with the anticancer drug doxorubicin were tested in a xenograft adenocarcinoma tumour model. The BRNPs were capable of loading anticancer drugs with high efficiency and capacity, prolonged the residence time of the drug in the circulation, exerted antiangiogenic effects, and significantly enhanced the efficiency of tumour-growth inhibition, especially in combination with exposure to external light.

**Figure 11.**
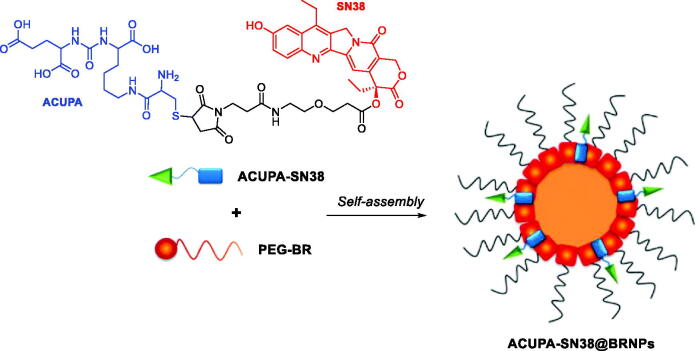
Illustration of the preparation of ACUPA-SN38@BRNPs in aqueous solution upon self-assembly^105^.

In another study, the group of Lee and Jon investigated the reported BRNPs as a tumour microenvironment (TME) responsive delivery carrier for small molecule drug conjugates (SMDCs)^105^. As a model SMDC, ACUPA-SN38 was synthesised by linking the prostate-specific membrane antigen (PSMA)-targeting ligand, that is ACUPA, to the chemotherapeutic agent, SN38 (Figure 11). ACUPA-SN38 was loaded into BRNPs using a film-formation and rehydration method. The resulting ACUPA-SN38@BRNPs exhibited ROS-mediated particle disruption and rapid release of the SMDC. BRNP-assisted delivery extended the half-life of the SMDC in the bloodstream, increased uptake by prostate cancer cells, and enhanced cytotoxicity, ultimately producing greater therapeutic efficacy than the parent SMDC in a tumour-bearing mouse model.

In a subsequent study, the same authors examined the potential of BRNPs as a therapy against IRI^106^. IRI is a major problem that may occur after liver transplantation and resection, and leads to an increase in inflammation and apoptosis that produces dysfunction of hepatic cells, organ rejection, and ultimately liver failure. Pro-inflammatory immune responses and increased oxidative stress damage the ischaemic tissue after restoration of blood flow. BRNPs were shown to exert a potent antioxidant and anti-apoptotic activity in primary hepatocytes exposed to hydrogen peroxide, a ROS precursor. In a mouse model of hepatic IRI, BRNP preconditioning exerted potent protective effects against hepatocellular injury by reducing oxidative stress, pro-inflammatory cytokine production, and recruitment of neutrophils. Notably, BRNPs also preferentially accumulated in IRI-induced inflammatory lesions.

**Figure 12.**
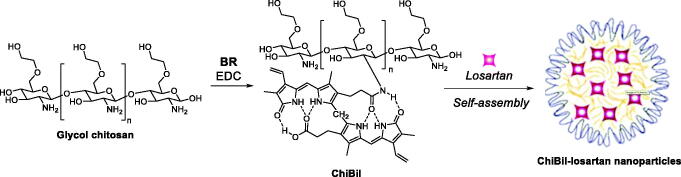
Synthesis of ChiBil conjugate and ChiBil-losartan formation upon self-assembly^107^.

A different type of nanoparticles including BR was designed by Surendran et al. in 2020 on the basis of the ROS induced rapid disruption of the BRNP nanostructures reported by the group of Lee and Jon^107^. In detail, the authors produced chitosan- bilirubin micelles (ChiBil), as ROS stimuli-responsive nanoparticle delivery system, carrying losartan for the treatment of hepatic fibrosis (Figure 12). The ChiBil amphiphilic conjugate self-assembles in water including losartan, an angiotensin receptor blocker that reduces hepatic fibrosis, as the therapeutic payload. The release characteristics of ChiBil- losartan were tested by ROS generation to confirm losartan release. Human hepatic stellate cell line LX2 was found to be the best *in vitro* model for the study. The reduction of hepatic stellate cell activation after treatment with ChiBil-losartan was analysed based on the expression of alpha-smooth muscle actin (α-SMA) in both *in vitro* and *in vivo* studies. Advanced liver fibrosis was induced in C3H/HeN mice using thioacetamide (TAA) via intraperitoneal injection and 10% ethanol (EtOH) in their drinking water. In addition, the hydroxyproline levels, histopathological evaluation, and mRNA quantification in the liver showed a decreased collagen content in the treated groups compared to that in the untreated control group. Macrophage infiltration studies and qPCR studies of inflammatory markers also proved the reduction of hepatic fibrosis in the treatment group. The intravenous administration of ChiBil-losartan resulted in decreased fibrosis in a TAA/EtOH-induced liver fibrosis mouse model. The *in vitro* and in vivo results suggest that the ROS stimuli-responsive ChiBil nanoparticles carrying losartan may be a potent therapeutic option for the treatment of hepatic fibrosis. The combined effect of losartan and bilirubin exhibited a decreased hepatic fibrosis both *in vitro* and *in vivo*.

The reduction of hepatic stellate cell activation after treatment with ChiBil-losartan was assessed both *in vitro* (LX2 cell lines) and in vivo (C3H/HeN mice). ChiBil-losartan nanoparticles showed to impair hepatic fibrosis progression and no toxicity in normal fibroblast, hepatocyte and LX-2 cell lines and mice.

**Figure 13.**
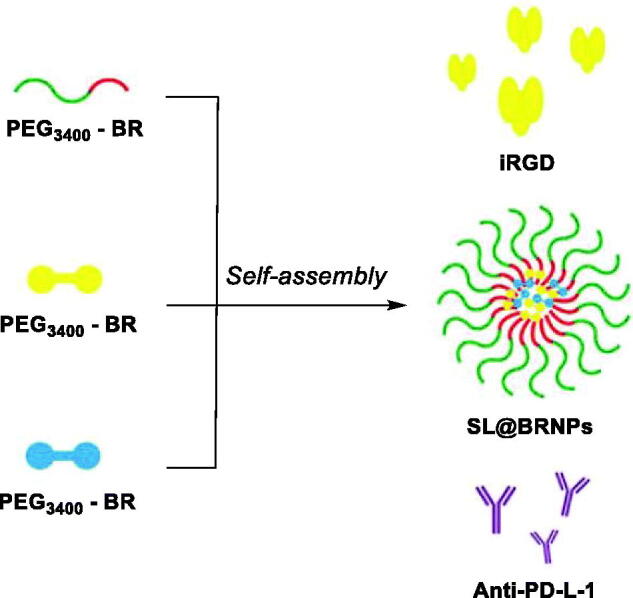
Illustration of PEGylated BR self-assembly to form d-SN38 and d-LND loaded nanoparticles, and their coadministration anti-PD-L1 and iRGD^108^.

In 2019, also Yang et al. produced ROS-responsive PEGylated BRNPs encapsulating two glutathione (GSH)-activatable anticancer drugs, that are dimer-7-ethyl-10-hydroxycamptothecin (d-SN38) and dimer-lonidamine (d-LND) (Figure 13)^108^. The amphiphilic PEG_3400_-BR efficiently entrapped hydrophobic chemotherapeutic drugs when self-assemblying and endowed nanoparticles with ROS responsiveness. The synthesis of dimeric drugs (d-SN38 and d-LND) with GSH-responsive bond greatly enhanced the encapsulation of drugs. iRGD, a tumour penetrating was coadministered with the SL@BRNPs to provide a better targeting and tumour permeating ability. After injection, iRGD promoted the enrichment of SL@BRNPs in tumour sites. d-SN38 and d-LND released from SL@BRNPs in the ROS-dependent manner. Subsequently, the dimeric drugs were cleaved upon contact with GSH. *In vitro* cell experiments demonstrated the superior toxicity of SL@ BRNPs/iRGD to 4T1 cells. Furthermore, the injected cocktail included programmed cell death ligand 1 antibody (anti-PD-L-1) used in immune-chemotherapy. The SL@BRNPs/iRGD + anti-PD-L1 therapy demonstrated superior tumour-targeting efficiency, strong suppression of breast cancer and its metastasis, while rarely affected normal tissue.

Another important research area in which BR recently gained much interest for a therapeutic application concerns intraportal pancreatic islet transplantation (IPIT), the only curative, non-invasive treatment for type-1 diabetes mellitus. However, 50–75% of islets fail to engraft at an early phase, representing a major impediment for successful IPIT. Alloantigen-specific immune-mediated destruction is a main cause for early islet failure, but non-specific inflammatory or innate immune responses were shown to play detrimental roles as well. In fact, islet cells suffer hypoxic stress due to their low expression of antioxidant enzymes^109^.

In 2010, Zhu and co-worker demonstrated for the first time that BR at an appropriate concentration or dose attenuates the cellular injury in rat insulinoma INS-1 cells induced by cytokines and, in a syngeneic rat model of IPIT, protects intraportally transplanted islets by inhibiting non-specific inflammatory response^110^. Nonetheless, in this study the authors identified a relatively narrow BR therapeutic window in respect to dosage.

In 2017, the research team of Lee and Jon tested their BRNPs (Figure 10) as an antioxidant and anti-inflammatory to prevent failure of pancreatic islet transplantation^111^. BRNPs were shown to protect islet cells not only from chemically induced oxidative stress by scavenging ROS, but also from activated macrophages by suppressing cytokine release. Importantly, *in vivo* experiments demonstrated that BRNP treatment can dramatically and significantly prolong islet graft survival compared to treatment with BR.

Again in 2017, Fullagar et al. proposed the encapsulation of bilirubin in pluronic 127–chitosan nanoparticle (nBR) to improve uptake by murine pancreatic islet cells and improve their resistance to hypoxic stress^112^. Pluronics are amphiphilic, triblock copolymers that form micelles in an oil-in-water emulsion, and pluronic F127 has been approved by the Food and Drug Administration (FDA) for use as a pharmaceutical ingredient. Pluronic F127–chitosan polymeric NPs demonstrated to be highly stable and effective in the delivery of BR, whose uptake was evaluated in INS-R3 cells and resulted to be improved via endocytosis. nBR resulted in preservation of normal islet architecture, prevention of central necrosis, and improved islet function. Nonetheless, cytotoxicity was detected at high doses which is speculated to be a result of the additive toxicity of BR and the NP themselves.

**Figure 14.**
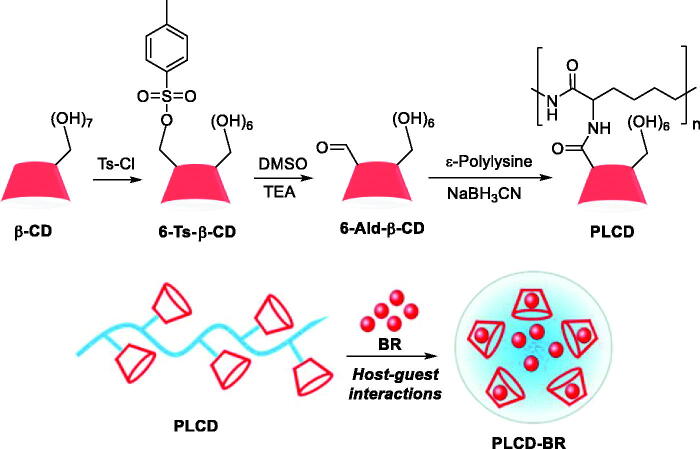
Synthetic route for PLCDs and supramolecular inclusion of BR to yield PLCD-BR^113^.

In 2020, Yao and co-workers designed a supramolecular carrier (PLCD) that could improve the solubility of BR and slowly release it to protect islets after cotransplantation^113^. PLCDs were synthesised by conjugating activated β-CDs to the side chain of ε-polylysine (PLL) and were thus loaded with BR through host − guest interactions to obtain the PLCD-BR inclusion complex (Figure 14). The constructed PLCD-BR system showed to protect islets from excessive oxidative stress and proinflammatory factors and improve islet survival and function *in vitro*. The diabetic mice cotransplanted with islets and PLCD-BR required the least time to achieve normoglycaemia among all tested groups, along with reduced inflammatory reactions and oxidative stress at the early stage of islet transplantation. Notably, PLCD-BR also contributed to the angiogenesis of the islet graft.

In 2020, Khurana et al. postulate the use of intravenous administration or inhalational delivery of BR nanomedicine (BNM) to combat systemic dysfunctions and complications associated with COVID-19, owing to the remarkable preclinical efficacy and optimistic results of various clinical studies of BR in a multitude of disorders^114^. Analysing the mechanism of the BR pharmacological effects, the author hypothesised the BR might halt the development of systemic inflammatory complications and cytokine storm detected with COVID-19. In conclusion, the authors deemed BNM a promising strategy for the pharmacotherapy of COVID-19.

## Conclusions

Here, we reviewed the two main aspects of bilirubin pharmacology, gathering literature data of applied pharmaceutical strategies adopted to: (i) reduce the plasma BR concentration to prevent neurotoxicity; (ii) produce a therapeutic effect based on the action of BR against a multitude of diseases. A *plethora* of pharmaceutical strategies, mostly based on hemoperfusion, have been proposed over the last decades to identify new valid, low risk alternatives to phototherapy and blood exchange transfusion for BR removal from plasma. In this context, the development of BR adsorbents with excellent mechanical properties, adsorption performance and hemocompatibility remains a considerable challenge. Additionally, platforms for BR blood removal upon enzymatic destruction were proposed, though not reaching a clinical application. Importantly, *per os* administration of drugs such as orlistat, PEG or bile acid derivatives was shown to induce hypobilirubinaemic effects both alone and in combination with phototherapy. On the other hand, a bunch of research studies dedicated to identify pharmaceutical strategies that promote the potent BR antioxidant, anti-inflammatory and immunomodulatory action for the treatment of several pathologies. Indeed, most studies proposed BRNPs based on the in solution self-assembly of PEGylated BR. BRNPs have been proposed: (i) as therapeutics against inflammatory diseases, IRI, and IPIT failure; (ii) as carriers of other drugs (mainly antitumor) based on BRNPs disruption upon exposure to ROS or external light. Relevant for the field, IPIT is main research area of BR therapeutic application to date. As a matter of fact, also encapsulation of BR in pluronic 127–chitosan nanoparticles and PLCD was investigated and shown to impair IPIT failure. To date, the therapeutic window of BR is not well-defined^49^^,^^115^. In fact, evidence indicates that the therapeutic concentrations of BR appear to be related to experimental design. Studies with cell culture models and perfused organs show a narrow therapeutic range, with effective doses in the range 10–20 µM and induced toxicity above 25 µM^42^^,^^116^. On the other hand, models involving systemic administration of BR in “whole animals” implicated higher doses to show beneficial effects with lack of apparent toxicity. In agreement with experimental observations in Gunn rats, serum BR concentrations >17 µM in human subjects with Gilbert’s Syndrome produced a profound protective effects against cardiovascular disease^117^^,^^118^.

## References

[1] Sticova E, Jirsa M. New insights in bilirubin metabolism and their clinical implications. World J Gastroenterol 2013;19:6398–407. 24151358 10.3748/wjg.v19.i38.6398 PMC3801310

[2] Čvorović J, Passamonti S. Membrane Transporters for Bilirubin and Its Conjugates: A Systematic Review. Front Pharmacol 2017;8:887. 29259555 10.3389/fphar.2017.00887 PMC5723324

[3] London IM, West R, Shemin D, Rittenberg D. On the origin of bile pigment in normal man. J Biol Chem 1950;184:351–8. 15422003

[4] Berk PD, Howe RB, Bloomer JR, Berlin NI. Studies of bilirubin kinetics in normal adults. J Clin Invest 1969;48:2176–90. 5824077 10.1172/JCI106184 PMC297471

[5] Gartung C, Matern S. Molecular regulation of sinusoidal liver bile acid transporters during cholestasis. Yale J Biol Med 1997;70:355–63. 9626756 PMC2589349

[6] Fisher MB, Paine MF, Strelevitz TJ, Wrighton SA. The role of hepatic and extrahepatic UDP-glucuronosyltransferases in human drug metabolism. Drug Metab Rev 2001;33:273–97. 11768770 10.1081/dmr-120000653

[7] van de Steeg E, Wagenaar E, van der Kruijssen CM, et al. Organic anion transporting polypeptide 1a/1b-knockout mice provide insights into hepatic handling of bilirubin, bile acids, and drugs. J Clin Invest 2010;120:2942–52. 20644253 10.1172/JCI42168 PMC2912192

[8] Ip S, Chung M, Kulig J, American Academy of Pediatrics Subcommittee on Hyperbilirubinemia, et al. An evidence-based review of important issues concerning neonatal hyperbilirubinemia. Pediatrics 2004;114:e130–e153. 15231986 10.1542/peds.114.1.e130

[9] Dani C, Pratesi S, Mannaioni G, Gerace E. Neurotoxicity of unconjugated bilirubin in neonatal hypoxic-ischemic brain injury in vitro. Front Pediatr 2021;9:659477. 33959576 10.3389/fped.2021.659477 PMC8093500

[10] Berska J, Bugajska J, Sztefko K. Newborns bilirubin concentration determined by different methods in relation to hematocrit and albumin level. J Med Biochem 2020;39:171–7. 33033449 10.2478/jomb-2019-0030 PMC7526020

[11] Dani C, Becciani S, Pratesi S. Changes in total serum bilirubin during phototherapy in late preterm and term infants with non-haemolytic hyperbilirubinemia. Early Hum Dev 2019;131:41–4. 30831388 10.1016/j.earlhumdev.2019.02.007

[12] Schiff D, Chan G, Poznansky MJ. Bilirubin toxicity in neural cell lines N115 and NBR10A. Pediatr Res 1985;19:908–11. 4047759 10.1203/00006450-198509000-00007

[13] Mustafa MG, Cowger ML, King TE. Effects of bilirubin on mitochondrial reactions. J Biol Chem 1969;244:6403–14. 4982202

[14] Diamond I, Schmid R. Oxidative phosphorylation in experimental bilirubin encephalopathy. Science 1967;155:1288–9. 6018655 10.1126/science.155.3767.1288

[15] Strumia E. [Effect of bilirubin on some hydrolases]. Boll Soc Ital Biol Sper 1959;35:2160–2. 13835320

[16] Flitman R, Worth MH. Inhibition of hepatic alcohol dehydrogenase by bilirubin. J Biol Chem 1966;241:669–72. 5908133

[17] Katoh R, Kashiwamata S, Niwa F. Studies on cellular toxicity of bilirubin: Effect on the carbohydrate metabolism in the young rat brain. Brain Res 1975;83:81–92.

[18] Greenfield S, Majumdar AP. Bilirubin encephalopathy: effect on protein synthesis in the brain of the Gunn rat. J Neurol Sci 1974;22:83–9. 4857425 10.1016/0022-510x(74)90056-2

[19] Majumdar AP. Bilirubin encephalopathy: effect on RNA polymerase activity and chromatin template activity in the brain of the Gunn rat. Neurobiology 1974;4:425–31. 4453356

[20] Sugatani J, Yamakawa K, Yoshinari K, et al. Identification of a defect in the UGT1A1 gene promoter and its association with hyperbilirubinemia. Biochem Biophys Res Commun 2002;292:492–7. 11906189 10.1006/bbrc.2002.6683

[21] Dubin IN, Johnson FB. Chronic idiopathic jaundice with unidentified pigment in liver cells; a new clinicopathologic entity with a report of 12 cases. Medicine (Baltimore) 1954;33:155–97. 13193360 10.1097/00005792-195409000-00001

[22] Rotor B, Manahan L, Florentin A. Familial non-hemolytic jaundice with direct van den Bergh reaction. Acta Medica Philippina 1948;5:37–49.

[23] Crigler JF, Najjar VA. Congenital familial nonhemolytic jaundice with kernicterus. Pediatrics 1952;10:169–80. 12983120

[24] Ritter JK, Yeatman MT, Ferreira P, Owens IS. Identification of a genetic alteration in the code for bilirubin UDP-glucuronosyltransferase in the UGT1 gene complex of a Crigler-Najjar type I patient. J Clin Invest 1992;90:150–5. 1634606 10.1172/JCI115829 PMC443074

[25] Jansen PL. Diagnosis and management of Crigler-Najjar syndrome. Eur J Pediatr 1999;158(Suppl 2):S89–S94. 10603107 10.1007/pl00014330

[26] Stokowski LA. Fundamentals of phototherapy for neonatal jaundice. Adv Neonatal Care 2006;6:303–12. 17208161 10.1016/j.adnc.2006.08.004

[27] Matas AJ, Sutherland DE, Steffes MW, Mauer SM, et al. Hepatocellular transplantation for metabolic deficiencies: decrease of plasms bilirubin in Gunn rats. Science 1976;192:892–4. 818706 10.1126/science.818706

[28] Arias IM. Chronic unconjugated hyperbilirubinemia without overt signs of hemolysis in adolescents and adults. J Clin Invest 1962;41:2233–45. 14013759 10.1172/JCI104682 PMC291158

[29] Gollan JL, Huang SN, Billing B, Sherlock S. Prolonged survival in three brothers with severe type 2 Crigler-Najjar syndrome. Ultrastructural and metabolic studies. Gastroenterology 1975;68:1543–55. 805737

[30] Gilbert A, Lereboullet P. La cholemie simple familiale. Semaine Medicale 1901;21:241–3.

[31] Owens D, Evans J. Population studies on Gilbert's syndrome. J Med Genet 1975;12:152–6. 1142378 10.1136/jmg.12.2.152 PMC1013257

[32] Monaghan G, McLellan A, McGeehan A, et al. Gilbert’ s syndrome is a contributory factor in prolonged unconjugated hyperbilirubinemia of the newborn. J Pediatr 1999;134:441–6. 10190918 10.1016/s0022-3476(99)70201-5

[33] Lee BK, Le Ray I, Sun JY, et al. Haemolytic and nonhaemolytic neonatal jaundice have different risk factor profiles. Acta Paediatr 2016;105:1444–50. Dec 27173507 10.1111/apa.13470

[34] Stocker R, Yamamoto Y, McDonagh AF, et al. Bilirubin is an antioxidant of possible physiological importance. Science 1987;235:1043–6. 3029864 10.1126/science.3029864

[35] Otero Regino W, Velasco H, Sandoval H. The protective role of bilirubin in human beings. Rev Col Gastroenterol 2009;24:357–364.

[36] Mayer M. Association of serum bilirubin concentration with risk of coronary artery disease. Clin Chem 2000;46:1723–7. 11067805

[37] Sedlak TW, Saleh M, Higginson DS, et al. Bilirubin and glutathione have complementary antioxidant and cytoprotective roles. Proc Natl Acad Sci USA 2009;106:5171–6. 19286972 10.1073/pnas.0813132106 PMC2664041

[38] Ziberna L, Martelanc M, Franko M, et al. Bilirubin is an endogenous antioxidant in human vascular endothelial cells. Sci Rep 2016;6:29240. 27381978 10.1038/srep29240 PMC4933905

[39] DiNicolantonio JJ, McCarty MF, O'Keefe JH. Antioxidant bilirubin works in multiple ways to reduce risk for obesity and its health complications. Open Heart 2018;5:e000914. 30364545 10.1136/openhrt-2018-000914 PMC6196942

[40] Hinds TD, Jr, Stec DE. Bilirubin safeguards cardiorenal and metabolic diseases: a protective role in health. Curr Hypertens Rep 2019;21:87. 31599366 10.1007/s11906-019-0994-z PMC6938163

[41] Ollinger R, Kogler P, Troppmair J, et al. Bilirubin inhibits tumor cell growth via activation of ERK. Cell Cycle 2007;6:3078–85. 18073533 10.4161/cc.6.24.5022

[42] Keshavan P, Schwemberger SJ, Smith DL, et al. Unconjugated bilirubin induces apoptosis in colon cancer cells by triggering mitochondrial depolarization. Int J Cancer 2004;112:433–45. 15382069 10.1002/ijc.20418

[43] Lacko M, Roelofs HM, Te Morsche RH, et al. Genetic polymorphism in the conjugating enzyme UGT1A1 and the risk of head and neck cancer. Int J Cancer 2010;127:2815–21. 21351260 10.1002/ijc.25296

[44] Zhong X, Liao Y, Chen X, et al. Abnormal serum bilirubin/albumin concentrations in dementia patients with a β deposition and the benefit of intravenous albumin infusion for Alzheimer's disease treatment. Front Neurosci 2020;14:859 33013289 10.3389/fnins.2020.00859 PMC7494757

[45] Horsfall LJ, Rait G, Walters K, et al. Serum bilirubin and risk of respiratory disease and death. JAMA 2011;305:691–7. 21325185 10.1001/jama.2011.124

[46] Yamaguchi T, Terakado M, Horio F, et al. Role of bilirubin as an antioxidant in an ischemia-reperfusion of rat liver and induction of heme oxygenase. Biochem Biophys Res Commun 1996;223:129–35. 8660358 10.1006/bbrc.1996.0857

[47] Liu Y, Li P, Lu J, et al. Bilirubin possesses powerful immunomodulatory activity and suppresses experimental autoimmune encephalomyelitis. J Immunol 2008;181:1887–97. 18641326 10.4049/jimmunol.181.3.1887

[48] Spetzler V, Goldaracena N, Kaths JM, et al. Elevated preoperative serum bilirubin improves reperfusion injury and survival postliver transplantation. Transplant Direct 2017;3:e187. 28795139 10.1097/TXD.0000000000000684 PMC5540625

[49] Dani C, Poggi C, Pratesi S. Bilirubin and oxidative stress in term and preterm infants. Free Radic Res 2019;53:2–7. 29768941 10.1080/10715762.2018.1478089

[50] Doğan M, Peker E, Kirimi E, et al. Evaluation of oxidant and antioxidant status in infants with hyperbilirubinemia and kernicterus. Hum Exp Toxicol 2011;30:1751–60. 21393350 10.1177/0960327111401638

[51] Dani C,E, Martelli G, Bertini M, et al. Plasma bilirubin level and oxidative stress in preterm infants. Arch Dis Child Fetal Neonatal Ed 2003;88:F119–123. 12598500 10.1136/fn.88.2.F119 PMC1721522

[52] Dani C, Masini E, Bertini G, et al. Role of heme oxygenase and bilirubin in oxidative stress in preterm infants. Pediatr Res 2004;56:873–7. 15470195 10.1203/01.PDR.0000145281.12853.9E

[53] Allam A, Ravikiran SR, Baliga BS, et al. Effect of conventional and LED phototherapy on the antioxidant-oxidant status in preterm neonates with jaundice. Indian Pediatr 2017;54:644–6. 28891476 10.1007/s13312-017-1127-x

[54] Dani C, Bertini G, Cecchi A, et al. Association between peak serum bilirubin and severity of respiratory distress syndrome in infants of less than 30 weeks' gestation”. J Perinat Med 2007;35:141–6. 17302512 10.1515/JPM.2007.023

[55] Zunszain PA, Ghuman J, McDonagh AF, Curry S. Crystallographic analysis of human serum albumin complexed with 4Z,15E-bilirubin-IXalpha. J Mol Biol 2008;381:394–406. 18602119 10.1016/j.jmb.2008.06.016 PMC2568863

[56] Czub MP, Handing KB, Venkataramany BS, et al. Albumin-based transport of nonsteroidal anti-inflammatory drugs in mammalian blood plasma. J Med Chem 2020;63:6847–62. 32469516 10.1021/acs.jmedchem.0c00225 PMC7902233

[57] Ghuman J, Zunszain PA, Petitpas I, et al. Structural basis of the drug-binding specificity of human serum albumin. J Mol Biol 2005;353:38–52. 16169013 10.1016/j.jmb.2005.07.075

[58] Ahlfors CE. Effect of ibuprofen on bilirubin-albumin binding. J Pediatr 2004;144:386–8. 15001951 10.1016/j.jpeds.2003.11.027

[59] Soligard HT, Nilsen OG, Bratlid D. Displacement of bilirubin from albumin by ibuprofen in vitro. Pediatr Res 2010;67:614–8. 20216106 10.1203/PDR.0b013e3181da7578

[60] Evoli S, Mobley DL, Guzzi R, Rizzuti B. Multiple binding modes of ibuprofen in human serum albumin identified by absolute binding free energy calculations. Phys Chem Chem Phys 2016;18:32358–68. 27854368 10.1039/c6cp05680f PMC5130592

[61] Paar M, Fengler VH, Rosenberg DJ, et al. Albumin in patients with liver disease shows an altered conformation. Commun Biol 2021;4:731. 34127764 10.1038/s42003-021-02269-w PMC8203801

[62] Berns M, Toennessen M, Koehne P, et al. Ibuprofen augments bilirubin toxicity in rat cortical neuronal culture. Pediatr Res 2009;65:392–6. 19127220 10.1203/PDR.0b013e3181991511

[63] Desfrere L, Thibaut C, Kibleur Y, et al. Unbound bilirubin does not increase during ibuprofen treatment of patent ductus arteriosus in preterm infants. J Pediatr 2012;160:258–64.e1. 21875717 10.1016/j.jpeds.2011.07.014

[64] Aoyagi Y, Ikenaka T, Ichida F. alpha-Fetoprotein as a carrier protein in plasma and its bilirubin-binding ability. Cancer Res 1979;39:3571–4. 89900

[65] Wong RJ, Ahmed A, Gish RG. Elevated alpha-fetoprotein: differential diagnosis - hepatocellular carcinoma and other disorders. Clin Liver Dis 2015;19:309–23. 25921665 10.1016/j.cld.2015.01.005

[66] Listowsky I, Gatmaitan Z, Arias IM. Ligandin retains and albumin loses bilirubin binding capacity in liver cytosol. Proc Natl Acad Sci USA 1978;75:1213–6. Mar 274712 10.1073/pnas.75.3.1213 PMC411440

[67] Wolkoff AW, Goresky CA, Sellin J, et al. Role of ligandin in transfer of bilirubin from plasma into liver. Am J Physiol 1979;236:E638–48. 375751 10.1152/ajpendo.1979.236.6.E638

[68] Erlinger S, Arias IM, Dhumeaux D. Inherited disorders of bilirubin transport and conjugation: new insights into molecular mechanisms and consequences. Gastroenterology 2014;146:1625–38. 24704527 10.1053/j.gastro.2014.03.047

[69] Keppler D. The roles of MRP2, MRP3, OATP1B1, and OATP1B3 in conjugated hyperbilirubinemia. Drug Metab Dispos 2014;42:561–5. 24459177 10.1124/dmd.113.055772

[70] Bockor L, Bortolussi G, Vodret S, et al. Modulation of bilirubin neurotoxicity by the Abcb1 transporter in the Ugt1-/- lethal mouse model of neonatal hyperbilirubinemia. Hum Mol Genet 2017;26:145–57. 28025333 10.1093/hmg/ddw375

[71] Bellarosa C, Bortolussi G, Tiribelli C. The role of ABC transporters in protecting cells from bilirubin toxicity. Curr Pharm Des 2009;15:2884–92. 19754365 10.2174/138161209789058246

[72] Brito MA, Palmela I, Cardoso FL, et al. Blood-brain barrier and bilirubin: clinical aspects and experimental data. Arch Med Res 2014;45:660–76. 25475697 10.1016/j.arcmed.2014.11.015

[73] Muchowski KE. Evaluation and treatment of neonatal hyperbilirubinemia. Am Fam Physician 2014;89:873–8. 25077393

[74] Lauer BJ, Spector ND. Hyperbilirubinemia in the newborn. Pediatr Rev 2011;32:341–9. 21807875 10.1542/pir.32-8-341

[75] Viggiano D, de Pascale E, Marinelli G, Pluvio C. A comparison among three different apheretic techniques for treatment of hyperbilirubinemia. J Artif Organs 2018;21:110–6. 28887736 10.1007/s10047-017-0986-1

[76] Chen X, Li L, Bai M, et al. Bilirubin adsorption for the treatment of severe hyperbilirubinemia after cardiac surgery: A retrospective cohort study. Int J Artif Organs 2021;8:391398821997841. 10.1177/0391398821997841 33678049

[77] Yu Y, He B, Gu H. Adsorption of bilirubin by amine-containing crosslinked chitosan resins. Artif Cells Blood Substit Immobil Biotechnol 2000;28:307–20. Jul 10928701 10.3109/10731190009119361

[78] Hirano S, Noishiki Y. The blood compatibility of chitosan and N-acylchitosans. J Biomed Mater Res 1985;19:413–7. 4055824 10.1002/jbm.820190406

[79] Wei H, Han L, Tang Y, et al. Highly flexible heparin-modified chitosan/graphene oxide hybrid hydrogel as a super bilirubin adsorbent with excellent hemocompatibility. J Mater Chem B 2015;3:1646–54. 32262437 10.1039/c4tb01673d

[80] Chen J, Yingda M, Lichun W, et al. Preparation of chitosan/SiO2-loaded graphene composite beads for efficient removal of bilirubin. Carbon 2019;143:352–61. e

[81] Li Z, Song X, Cui S, et al. Fabrication of macroporous reduced graphene oxide composite aerogels reinforced with chitosan for high bilirubin adsorption. RSC Adv 2018;8:8338–48. 10.1039/c8ra00358k PMC9078524 35542023

[82] Ando K, Shinke K, Yamada S, et al. Fabrication of carbon nanotube sheets and their bilirubin adsorption capacity. Colloids Surf B Biointerfaces 2009;71:255–9. 19327971 10.1016/j.colsurfb.2009.02.017

[83] Nagyvary JJ. Method for treating hyperbilirubinemia, 1981, US4363801A.

[84] Crini G. Review: A History of Cyclodextrins. Chem Rev 2014;114:10940–75. 25247843 10.1021/cr500081p

[85] Kano K, Yoshiyasu K, Hashimoto SJ. Enantioselective complexation of bilirubin with cyclodextrins and non-cyclic oligosaccharides. J Chem Soc Chem Commun. 1988;801–2.

[86] Wang Z, Cao Y, Wei H, et al. Bilirubin adsorption properties of water-soluble adsorbents with different cyclodextrin cavities in plasma dialysis system. Colloids Surf B Biointerfaces 2012;90:248–53. 22037476 10.1016/j.colsurfb.2011.10.006

[87] Gemelli C, Cuoghi A, Magnani S, et al. Removal of bilirubin with a new adsorbent system: in vitro kinetics. Blood Purif 2019;47:10–5. 30219813 10.1159/000492378

[88] Lavin A, Sung C, Klibanov AM, Langer R. Enzymatic removal of bilirubin from blood: a potential treatment for neonatal jaundice. Science 1985;230:543–5. 4048947 10.1126/science.4048947

[89] Kimura M, Matsumura Y, Miyauchi Y, Maeda H. A new tactic for the treatment of jaundice: an injectable polymer-conjugated Bilirubin oxidase. Proc Soc Exp Biol Med 1988;188:364–9. 2455905 10.3181/00379727-188-42747

[90] Kimura M, Matsumura Y, Konno T, et al. Enzymatic removal of bilirubin toxicity by bilirubin oxidase in vitro and excretion of degradation products in vivo. Proc Soc Exp Biol Med 1990;195:64–9. 2399262 10.3181/00379727-195-43119

[91] Sarkar S, Makhal A, Baruah S, et al. Nanoparticle-Sensitized Photodegradation of Bilirubin and Potential Therapeutic Application. J. Phys. Chem. C 2012; 116:9608–15.

[92] Nishioka T, Hafkamp AM, Havinga R, et al. Orlistat treatment increases fecal bilirubin excretion and decreases plasma bilirubin concentrations in hyperbilirubinemic Gunn rats. J Pediatr 2003;143:327–34. 14517515 10.1067/s0022-3476(03)00298-1

[93] Hafkamp AM, Havinga R, Ostrow JD, et al. Novel kinetic insights into treatment of unconjugated hyperbilirubinemia: phototherapy and orlistat treatment in Gunn rats. Pediatr Res 2006;59:506–12. 16549520 10.1203/01.pdr.0000203180.79636.98

[94] Cuperus FJ, Iemhoff AA, van der Wulp M, et al. Acceleration of the gastrointestinal transit by polyethylene glycol effectively treats unconjugated hyperbilirubinaemia in Gunn rats. Gut 2010;59:373–80. 19893023 10.1136/gut.2009.183921

[95] Klaschik E, Nauck F, Ostgathe C. Constipation-modern laxative therapy. Support Care Cancer 2003;11:679–e85. 14505158 10.1007/s00520-003-0525-x

[96] Cuperus FJ, Hafkamp AM, Havinga R, et al. Effective treatment of unconjugated hyperbilirubinemia with oral bile salts in Gunn rats. Gastroenterology 2009;136:673–82.e1. 19027011 10.1053/j.gastro.2008.10.082

[97] van der Schoor LWE, Verkade HJ, Bertolini A, et al. Potential of therapeutic bile acids in the treatment of neonatal Hyperbilirubinemia. Sci Rep 2021;11:11107. 34045606 10.1038/s41598-021-90687-5 PMC8160219

[98] Bakrania B, Bulmer AC, Wagner KH, et al. Bilirubin acts as a multipotent guardian of cardiovascular integrity: more than just a radical idea. Am J Physiol Heart Circ Physiol 2018;315:H429–H447. 29600900 10.1152/ajpheart.00417.2017

[99] Kundur AR, Bulmer AC, Singh I. Unconjugated bilirubin inhibits collagen induced platelet activation. Platelets 2014;25:45–50. 23406485 10.3109/09537104.2013.764405

[100] Kundur AR, Singh I, Bulmer AC. Bilirubin, platelet activation and heart disease: a missing link to cardiovascular protection in Gilbert's syndrome? Atherosclerosis 2015;239:73–84. 25576848 10.1016/j.atherosclerosis.2014.12.042

[101] Pennell EN, Wagner KH, Mosawy S, et al. Acute bilirubin ditaurate exposure attenuates ex vivo platelet reactive oxygen species production, granule exocytosis and activation. Redox Biol 2019;26:101250. 31226648 10.1016/j.redox.2019.101250 PMC6586953

[102] Lee Y, Kim H, Kang S, et al. Bilirubin Nanoparticles as a Nanomedicine for Anti-inflammation Therapy. Angew Chem Int Ed Engl 2016;55:7460–3. 27144463 10.1002/anie.201602525

[103] Kim DE, Lee Y, Kim M, et al. Bilirubin nanoparticles ameliorate allergic lung inflammation in a mouse model of asthma. Biomaterials 2017;140:37–44. 28624706 10.1016/j.biomaterials.2017.06.014

[104] Lee Y, Lee S, Lee DY, et al. Multistimuli-responsive bilirubin nanoparticles for anticancer therapy. Angew Chem Int Ed Engl 2016;55:10676–80. 27485478 10.1002/anie.201604858

[105] Lee S, Lee Y, Kim H, et al. Bilirubin nanoparticle-assisted delivery of a small molecule-drug conjugate for targeted cancer therapy. Biomacromolecules 2018;19:2270–7. 29712433 10.1021/acs.biomac.8b00189

[106] Kim JY, Lee DY, Kang S, et al. Bilirubin nanoparticle preconditioning protects against hepatic ischemia-reperfusion injury. Biomaterials 2017;133:1–10. Jul 28414974 10.1016/j.biomaterials.2017.04.011

[107] Surendran SP, Thomas RG, Moon MJ, et al. A bilirubin-conjugated chitosan nanotheranostics system as a platform for reactive oxygen species stimuli-responsive hepatic fibrosis therapy. Acta Biomater 2020;116:356–67. 32927089 10.1016/j.actbio.2020.09.014

[108] Yang X, Hu C, Tong F, et al. Tumor microenvironment-responsive dual drug dimer-loaded PEGylated bilirubin nanoparticles for improved drug delivery and enhanced immune-chemotherapy of breast cancer. Adv. Funct. Mater 2019; 29:1901896.

[109] Yin D, Ding JW, Shen J, et al. Liver ischemia contributes to early islet failure following intraportal transplantation: benefits of liver ischemic-preconditioning. Am J Transplant 2006;6:60–8. 16433757 10.1111/j.1600-6143.2005.01157.x

[110] Zhu H, Wang J, Jiang H, et al. Bilirubin protects grafts against nonspecific inflammation-induced injury in syngeneic intraportal islet transplantation. Exp Mol Med 2010;42:739–48. 20881452 10.3858/emm.2010.42.11.075 PMC2992853

[111] Kim MJ, Lee Y, Jon S, Lee DY. PEGylated bilirubin nanoparticle as an anti-oxidative and anti-inflammatory demulcent in pancreatic islet xenotransplantation. Biomaterials 2017;133:242–52. 28448818 10.1016/j.biomaterials.2017.04.029

[112] Fullagar B, Rao W, Gilor C, et al. Nano-encapsulation of bilirubin in pluronic F127-chitosan improves uptake in β cells and increases islet viability and function after hypoxic stress. Cell Transplant 2017;26:1703–15. 29251115 10.1177/0963689717735112 PMC5753985

[113] Yao Q, Huang ZW, Zhai YY, Yue M, et al. Localized controlled release of bilirubin from β-cyclodextrin-conjugated ε-polylysine to attenuate oxidative stress and inflammation in transplanted islets. ACS Appl Mater Interfaces 2020;12:5462–75. 31927945 10.1021/acsami.9b18986

[114] Khurana I, Allawadhi P, Khurana A, et al. Can bilirubin nanomedicine become a hope for the management of COVID-19? Med Hypotheses 2021;149:110534 33640714 10.1016/j.mehy.2021.110534 PMC7881296

[115] Adin CA. Bilirubin as a therapeutic molecule: challenges and opportunities. Antioxidants (Basel) 2021;10:1536. 34679671 10.3390/antiox10101536 PMC8532879

[116] Khan NM, Poduval TB. Immunomodulatory and immunotoxic effects of bilirubin: Molecular mechanisms. J Leukoc Biol 2011;90:997–1015. 21807743 10.1189/jlb.0211070

[117] Barabas K, Milner R, Farese J, et al. Hyperbilirubinemia's protective effect against cisplatin nephrotoxicity in the Gunn rat. Anticancer Drugs 2008;19:495–502. 18418216 10.1097/CAD.0b013e3282fdc391 PMC4356238

[118] Lin J-P, O’Donnell CJ, Schwaiger JP, et al. Association between the UGT1A1*28 allele, bilirubin levels, and coronary heart disease in the Framingham Heart Study. Circulation 2006;114:1476–81. 17000907 10.1161/CIRCULATIONAHA.106.633206

